# Guidelines for Reporting Medical Research: A Critical Appraisal

**DOI:** 10.1155/2016/1346026

**Published:** 2016-03-22

**Authors:** Mathilde Johansen, Simon Francis Thomsen

**Affiliations:** ^1^Department of Biomedical Sciences, Faculty of Health and Medical Sciences, University of Copenhagen, 2200 Copenhagen N, Denmark; ^2^Department of Dermatology, Bispebjerg Hospital, 2400 Copenhagen NV, Denmark

## Abstract

As a response to a low quality of reporting of medical research, guidelines for several different types of study design have been developed to secure accurate reporting and transparency for reviewers and readers from the scientific community. Herein, we review and discuss the six most widely accepted and used guidelines: PRISMA, CONSORT, STROBE, MOOSE, STARD, and SPIRIT. It is concluded that the implementation of these guidelines has led to only a moderate improvement in the quality of the reporting of medical research. There is still much work to be done to achieve accurate and transparent reporting of medical research findings.

## 1. Introduction

According to the GRADE (Grading of Recommendations Assessment, Development and Evaluation) methodology, study designs in medical research can be hierarchically grouped based on their level of evidence and their strength of recommendation of clinical interventions (Table 1) [1]. According to this hierarchy, systematic reviews and meta-analyses of randomized controlled trials (RCTs) rank first followed by individual RCTs. Below in the hierarchy are nonrandomized trials and observational study designs such as cohort studies and case-control studies, whereas case studies and expert opinions, the so-called anecdotal evidence, are ranked at the bottom, although they might still have high impact on clinical decision-making.

Well-designed studies are, however, not sufficient to ensure transparency in medical research. It is the presentation of evidence that is of great importance in the published scientific article. Notably, to be able to judge the merit and potential impact of a scientific study reported in a journal article, the reader must know exactly how the study was done and what was found. It is essential to easily appraise whether or not the accomplished research is to have any influence on healthcare. Researchers use articles as guidance on how to elaborate a trial and to see if their results have any effect on their own research. Clinicians use scientific articles to make out the best treatment of a patient, and finally, government healthcare providers and public stakeholders utilize them to guide overall preventive and treatment strategies.

In order to ensure this transparency and accuracy of reporting medical research, several guidelines have been gradually introduced [2–4]. Currently, the EQUATOR (Enhancing the QUAlity and Transparency Of health Research) network [5] has registered 256 guidelines pertaining to various topics within medical research. However, as early as in 1938, a textbook was published with a chapter about how medical research should be published [6]. Of note, it was stated that the importance of reporting the results correctly was not only for the very critical readers' satisfaction, but also for the sake of keeping the value in the results [6]. In 1988, the International Committee of Medical Journal Editors included a statement in their guidelines to authors stating that the statistical methods should be described so thoroughly that a reviewer could verify the results reported [2]. In 1994, the first attempt to create a reporting guideline was made, which eventually laid the groundwork for the development of the Consolidated Standards of Reporting Trials (CONSORT) statement in 1996 [2].

Herein, we review the six most widely used guidelines for reporting medical research findings (PRISMA, CONSORT, STROBE, MOOSE, STARD, and SPIRIT; see meaning of abbreviations below, Table 2) and investigate how well they are applied in the medical literature. Furthermore, we clarify the advantages and disadvantages of using guidelines for reporting medical research findings.

## 2. Description of the Specific Guidelines

### 2.1. PRISMA (http://www.prisma-statement.org/)

PRISMA (Preferred Reporting Items for Systematic Reviews and Meta-analyses) is developed for the reporting of systematic reviews and meta-analyses [7]. In 1987, two independent studies of adequacy in reporting of systematic reviews and meta-analyses found that reporting was generally insufficient and did not fulfill the anticipated criteria [8, 9]. In 1996, an update to one of the studies was made, and no significant improvement was found, which led to the formulation of the QUOROM (QUality Of Reporting Of Meta-analyses) statement [10]. QUOROM was updated to PRISMA in 2005 due to the fact that there had been some changes in the science of systematic reviews concerning conceptual and practical advances. In the process of updating the guideline, it also aimed to improve the consistency throughout the systematic review report. Scientific authors, methodologists, medical editors, clinicians, and a consumer participated in the update.

The PRISMA statement consists of a checklist of 27 items, which are divided into the following categories: title, abstract, introduction, methods, results, discussion, and funding. The PRISMA statement also endorses the use of a flow diagram. The aim of this statement is to increase transparency and to improve the reporting of systematic reviews and meta-analyses. Furthermore, the statement is useful when critically appraising published systematic reviews.

In 2013, only about 30% of medical journals recommended the PRISMA statement to their authors [11]. In the same year, an examination of systematic reviews from 2012 showed that articles published in journals that endorsed PRISMA included, on average, 90.1% of the items, whereas 85.3% of the items were present in articles from journals that did not endorse the PRISMA statement [11]. In particular, there was a significantly higher rate of adherence to item number 17 (“study selection”) of PRISMA (100.0% versus 63.3%). Furthermore, there was an increase from 83.1% to 90.1% in reported items from before the creation of the statement until 2012 [11]. The study also showed that there had been a significant increase in methodological quality of published studies after the introduction of PRISMA.

### 2.2. CONSORT (http://www.consort-statement.org/)

CONSORT (Consolidated Standards of Reporting Trials) is a guideline for reporting randomized controlled trials, of which the latest version is from 2010 [3]. The first CONSORT statement was developed in 1996 as empirical evidence implied that authors reported trials badly due to the possible association with bias [12]. In 2001, a revision was made, followed by a second revision in 2010, which was based on accumulated experience. Empirical evidence to support the statement is located in a database, which is generated on the basis of more than 700 studies [3]. The CONSORT workgroup that keeps the item checklist up to date consists of biomedical editors, clinical trialists, statisticians, and epidemiologists. With this constellation, the CONSORT executive strives to make a balance between established and emerging researchers.

The CONSORT consists of a 25-item checklist and is divided into subcategories: title and abstract, introduction, methods, results, discussion, and other information. This structure is intended to promote complete reporting and transparent research. Indirectly, this structure also influences trial design, conduction, and publication of the trial. This is the foremost aim of the structure, as it will prevent inadequately designed trials from being published. Furthermore, CONSORT consists of a flow diagram, which enables the acquirement of a general view of the phases that patients in the trial go through [2].

When the edited version of CONSORT was published, more than 400 journals supported the CONSORT statement [3]. CONSORT is not made to be followed rigidly, which leaves room to abide by the traditions in the specific research field, journal style, editorial directions, and also, whenever possible, the authors' preferences. It has been stated that the quality of reporting trials has improved [13]. Particularly, a study conducted two years after the development of CONSORT showed an improvement in the reporting attributable to the statement [14]. The study compared randomized controlled trials published before CONSORT (1994) and after CONSORT (1998). The study was conducted in a way where the items from the checklist were modified and expanded so that multiple items were listed as separate, which led to 40 items. The improvement was seen in the reporting of items from 23.4 before CONSORT with a mean change of 3.7, whereas a decrease of 22% was seen for how often unclear reporting of allocation concealment took place. This mean change in the reporting of items from the checklist is, as mentioned above, still not adequate, when keeping in mind that, to fulfill all the criteria and secure transparency, authors should report all 40 items. However, it is important to remember that the study was conducted only two years after the implementation of the guideline.

### 2.3. STROBE (http://www.strobe-statement.org/)

STROBE (STrengthening the Reporting of OBservational studies in Epidemiology) was developed in 2004 [15]. It is used as a guideline for reporting observational studies, specifically cohort, case-control, and cross-sectional studies. The guideline arose from the aspiration that this type of research should be transparently reported to allow the reader to follow what was planned, done, and found and which conclusions were drawn. In a series of examinations, it was found that the reporting of these topics was insufficient for observational studies [16, 17]. It was discovered that the specification of potentially confounding variables often was missing, which was the same case for the explanation of how, for example, a control and case group were selected. A further aim was to provide guidance on how observational research could be reported accurately. The inspiration for STROBE stemmed from the CONSORT statement, and a group of methodologists, epidemiologists, statisticians, practitioners, and journal editors developed the STROBE statement.

The STROBE statement consists of a 22-item checklist under the following headings: title and abstract, introduction, methods, results, discussion, and other information. 18 of the items are identical for the three types of studies, while four items differ. As for CONSORT, the guideline is not meant to be followed strictly, and the presentation of information should depend on the journal style, the authors' preferences, and the traditions in the research area.

STREGA (STrengthening the REporting of Genetic Association studies) is an extension to STROBE [18]. Whereas STROBE is used for observational studies (analytical epidemiology), STREGA is used for genetic association studies. It is the hope that extensions are made to STROBE to cover other specific topic areas as well.

### 2.4. MOOSE (http://www.consort-statement.org/downloads)

MOOSE (Meta-analysis Of Observational Studies in Epidemiology) is a guideline used for reporting meta-analyses of observational studies [19]. In 1997, a workshop was held to design a guideline to improve the usefulness of epidemiological meta-analyses. It was discovered that an increasing diversity and variability existed in the reporting of these meta-analyses. The result was a 35-item checklist with the following headings: background, search strategy, methods, results, discussion, and conclusion. The aim of this guideline was to improve the usefulness of epidemiological meta-analyses by showing more clearly what was done, who did it, and why it was done in order to help researchers reach this goal.

### 2.5. STARD (http://www.stard-statement.org/)

STARD (STAndards for the Reporting of Diagnostic accuracy studies) is intended for reporting studies of diagnostic or prognostic accuracy [4]. A number of articles in four medical journals between 1978 and 1993 laid the foundations for a survey of studies of diagnostic accuracy that showed that the methodological quality was poor or at best mediocre. The results of these studies turned out to be hard to evaluate because key elements of design, conduct, and analysis were missing in the majority. It was shown, when compared with other studies, that specific design features were associated with biased estimates of diagnostic accuracy [20]. Consequently, a group was assembled in 1999 to discuss these low standards. The intention of the group was to create a guideline inspired by CONSORT, with the goal of improving the quality in reporting of studies of diagnostic accuracy. The group stated that the detection of potential biases would be increased with complete and accurate reporting combined with the possibility of generalizing and applying the results to other cases. Finally, the checklist concluded on 25 items under the following headlines: title/abstract/keywords, introduction, methods, results, and discussion. A flow diagram adds to the checklist bringing information about the method used for patient recruitment and information about in which order the tests had been carried out.

In 2008, a review based on two different studies, which evaluated the quality of reporting after STARD's development, concluded that the intended effect of STARD was not yet achieved [21]. The review offered a number of suggestions as to why this was the case. One suggestion was a slow adoption rate in the medical journals, which was in line with what other studies have concluded [22]. A second suggestion was the way the journals described how the authors should apply the guideline. This varied greatly between different journals and must have caused some confusion when one journal strictly advocated the use of the statement while others advised that it should only be consulted [21, 23].

A study conducted in 2013 examined the different evaluations conducted since STARD was introduced [22]. It showed an overall improvement in the quality of the reporting of diagnostic accuracy studies; nevertheless, the studies were still hampered by lack of quality. The guidelines had been followed by many, but some of the very important items from the list were still missing. Important items such as “blinding of readers” and “methods for calculating test reproducibility” were omitted, thus resulting in the possibility of biased results. Notably, many researchers still did not apply the flow diagram. Many of the studies included in the review still recommended the use of STARD as it resulted in better reporting. It is also worth remembering that many of these studies were conducted shortly after the introduction of STARD and therefore the implementation time has been short. A recent report, which supports the above results, compared studies conducted before, shortly after, and 10 years after the implementation of STARD [24]. The main finding was an overall increase of 3.4 reported items from the STARD checklist from before the implementation compared with 10 years after. In contrast to previous evaluations of STARD, this report also showed a significant increase in the use of the flowchart. Moreover, this study suggested that some effort should be put into the education of peer reviewers and journal editors in order to endorse the use of STARD.

### 2.6. SPIRIT (http://www.spirit-statement.org/)

SPIRIT (Standard Protocol Items: Recommendations for Interventional Trials) was created in 2007 for the reporting of scientific trial protocols [25]. The need for such a guideline was evident through a systematic review, which found that many protocols for randomized trials lacked information on important components of the trial, such as primary outcome, treatment allocation methods, and the use of blinding (masking) [26]. It was examined that these shortcomings could lead to inadequate reporting, poor trial conduct, and protocol amendments.

In the development of SPIRIT, a team of trial investigators, healthcare professionals, methodologists, statisticians, trial coordinators, journal editors, and representatives from the research ethics community, industrial and nonindustrial funders, and regulatory agencies worked together. They created a statement that clarified the requirements for protocols in clinical trials, that is, a list that included 33 items divided into the following domains: administrative information, introduction, methods, ethics and dissemination, and appendices. Furthermore, it was advised that the protocol kept a format, which included a table of contents, section headings, glossary, and list of references. The format of SPIRIT has incorporated some items and inspiration from CONSORT in order to enable an easier transition from a SPIRIT-based protocol to a final CONSORT-based report. It was hoped that SPIRIT would promote transparency as well as an adequate description of how the trial was planned. Furthermore, it was hoped that, by improving the completeness of protocols, queries to investigators about unclear information would be reduced, thereby leading to an increase in efficiency. In addition, it might ensure the requisite information for critical appraisal and trial interpretation of the protocols.

SPIRIT helps to “lock” the protocol in conjunction with mandatory registration at web domains such as https://clinicaltrials.gov/ where studies have to be registered before commencement to ensure transparency in the execution and reporting of the study. By applying the SPIRIT statement to “lock” the protocol, studies should not, for example, switch endpoints so that a secondary endpoint is lifted to become primary in the case where the primary outcome does not meet the prespecified level of statistical significance.

## 3. Discussion

The publication of the CONSORT statement initiated a cascade of changes in the reporting of medical research in scientific journals. As outlined above, this has had great impact on the quality of reporting of various types of medical research. Specifically, it has been emphasized that changes have to be made on how the guidelines are applied [23]. Still, the introduction of these various reporting guidelines has not yet secured complete transparency and accurate reporting, mainly because they are not followed as rigorously as was intended. In particular, it is argued that neither journal editors nor peer reviewers want to take full responsibility for checking whether guidelines are adhered to. To attend to this problem, academic employees at the editorial offices of major journals need to secure that submitted manuscripts adhere to the relevant guideline before they are sent to the associate editors and to external peer review. This is costly and will to some extent delay the review process but will make manuscripts more suitable for publication should they meet other relevant criteria for scientific merit. Another way of securing adherence to reporting guidelines is the instruction of the Committee on Publication Ethics (COPE) Ethical Guidelines for Peer Reviewers [27] that sets forth standards for peer reviewing ethics. The COPE guideline is endorsed by major journals and sets out the basic principles and standards to which peer reviewers should adhere during the peer review process. Finally, predefined strategies for updating and evaluating the various guidelines have not been made. Ideally, guidelines should be rigorously evaluated and updated regularly based on accumulated evidence.

Undoubtedly, there are barriers of communicating efficiently the advantages and disadvantages of compliance to guidelines. When reporting research findings, authors are expected to make sure that the correct guideline is being applied. On journals' websites, which guideline they stipulate and how they want them to be followed should be stated. This varies greatly between journals [21, 23]. Furthermore, readers of any kind have to familiarize themselves with the guideline by which the article is written. In this regard, the EQUATOR network offers some support through courses for authors, reviewers, and editors [13]. However, it has been argued that editors find it a practical burden and out of their competence to check all submitted articles, and most editors do not want to be the gatekeepers of the correct use of reporting guidelines [23]. To this end, the obligation of adhering to publication guidelines relies solely on the (group of) author(s).

So why do the reported findings still lack transparency and accuracy with all these guidelines? Both the CONSORT statement and an evaluating article state that the guidelines might encourage some authors to fabricate spurious information in order to comply with the statement [2, 3]. Furthermore, authors might be limited to article word counts and as a consequence feel the necessity to leave out important items that fulfill the demands of the journal [28]. Certain authors might be reluctant to comply with some guidelines, as they feel deprived of their liberty in the research because the study has to match perfectly the guideline assigned to that type of study [23]. If this is the case, then the solution for more transparent reporting might not be to follow the guidelines more precisely but rather to find another way for authors to apply guidelines, which would help them express their message more clearly and not limit them.

Another implication of reporting guidelines is the missing reporting of research. Some research is currently not being published because the application of the guidelines makes it particularly apparent that a study has limitations or does not conclude desired results [2]. If only articles that adhere to reporting guidelines are published, a distorted picture on the field will be created (publication bias). In this instance, reporting guidelines constitute a barrier that prevents inferior research from being published in high-ranking journals and in turn from being cited (high-impact journal articles cite primarily articles from the same or other high-impact journals). However, many low- to middle-impact journals allow publication of research articles that do not adhere to reporting guidelines. In combination with the open access strategy of many of these journals, this mitigates the publication bias introduced by the inappropriate or inefficient use of relevant guidelines.

## 4. Conclusion

The purpose of having reporting guidelines in medical research is to create a manual for the authors to follow, which should lead to total transparency, accurate reporting, and easier assessment of the validity of reported research findings. This goal has been reached to some degree, but it is still necessary to be critical when appraising any research article. It might be time for editors, authors, and reviewers to assemble and figure out how to best use and recommend the various reporting guidelines.

## Figures and Tables

**Table 1:** Hierarchy of evidence.

Study type	Level of evidence	Strength of recommendation
Systematic reviews and meta-analyses of randomized controlled trials	1a	A
Randomized controlled trials	1b	

Nonrandomized controlled trials	2a	B
Cohort studies	2b	

Case-control studies	3	C

Case studies, expert opinions	4	D

Diagnostic studies	—	—

**Table 2:** Guidelines for reporting medical research.

Guideline	Year of introduction	Year of change	Study type	Sections	Number of items	Flow diagram
PRISMA	1996 (QUORUM)	2005	Systematic reviews and meta-analyses	Title; abstract; introduction; methods; results; discussion; funding	27	Yes

CONSORT	1996	2001 and 2010	Parallel group randomized controlled trials	Title and abstract; introduction; methods; results; discussion; other information	25	Yes

STROBE	2004		Observational studies in epidemiology	Title and abstract; introduction; methods; results; discussion; other information	22	No

MOOSE	1997		Meta-analyses of observation studies in epidemiology	Background; search strategy; methods; results; discussion; conclusion	35	No

STARD	2000		Studies of diagnostic accuracy	Title/abstract/keywords; introduction; methods; results; discussion	25	Yes

SPIRIT	2007	2013	Standard protocol items for clinical trials	Administrative information; introduction; methods; ethics and dissemination; appendices	33	No, but template with recommended content of enrolment, interventions, and assessments
